# Neonatal Acute Kidney Injury: A Survey of Perceptions and Management Strategies Amongst Pediatricians and Neonatologists

**DOI:** 10.3389/fped.2019.00553

**Published:** 2020-01-14

**Authors:** Sidharth Kumar Sethi, Gopal Agrawal, Sanjay Wazir, Smriti Rohatgi, Arpana Iyengar, Ronith Chakraborty, Rahul Jain, Nikhil Nair, Rajiv Sinha, Raktima Chakrabarti, Deepak Kumar, Rupesh Raina

**Affiliations:** ^1^Department of Pediatric Nephrology, Medanta the Medicity, Gurgaon, India; ^2^Department of Pediatrics and Neonatology, Cloudnine Hospital, Gurgaon, India; ^3^Department of Pediatrics, St. John's National Academy of Health Sciences, Bengaluru, India; ^4^Akron Nephrology Associates/Cleveland Clinic Akron General Medical Center, Akron, OH, United States; ^5^Saint Ignatius High School, Cleveland, OH, United States; ^6^Department of Chemistry, Case Western Reserve University, Cleveland, OH, United States; ^7^Department of Pediatric Medicine, Institute of Child Health, Kolkata, India; ^8^Department of Pediatrics, Case Western Reserve University, Cleveland, OH, United States; ^9^Department of Nephrology, Akron Children's Hospital, Akron, OH, United States

**Keywords:** neonatal, acute kidney injury, AKI, dialysis, preterm, survey

## Abstract

**Background:** Neonatal Acute Kidney Injury (AKI) occurs in 40–70% of critically ill newborn infants and is independently associated with increased morbidity and mortality. Understanding the practice patterns of physicians (neonatologists and pediatricians), caring for neonates in India is important to optimize care and outcomes in neonatal AKI.

**Aim:** The aim of this study was to identify differences in physician's perception and practice variations of diagnosis, management, and follow-up of newborn infants with AKI in India.

**Methods:** An online survey of neonatologists and pediatricians in India caring for newborn infants with AKI.

**Results:** Out of 800 correspondents, 257 (135 neonatologists and 122 pediatricians) completed the survey, response rate being 32.1%. Resources available to the respondents included level III NICU (59%), neonatal surgery (60%), dialysis (11%), and extracorporeal membrane oxygenation (ECMO, 3%). Most respondents underestimated the risk of AKI due to various risk factors such as prematurity, asphyxia, sepsis, cardiac surgery, and medications. Less than half the respondents were aware of the AKIN or KDIGO criteria, which are the current standard criteria for defining neonatal AKI. Only half of the respondents were aware of the risk of CKD in preterm neonates and nearly half were unaware of the need to follow up with a pediatric nephrologist.

**Conclusions:** Similar to other regions worldwide, there exists a knowledge gap in early recognition, optimal management and follow up of newborn infants with AKI amongst Indian physicians.

## Introduction

Neonatal Acute Kidney Injury (AKI) is common amongst the Neonatal Intensive Care Unit (NICU) infants and is a major contributor of neonatal mortality and morbidity (1, 2). Although precise prevalence is unknown, reported frequency of neonatal AKI ranges from 6 to 24% (3, 4). Since nephrogenesis is incomplete before 36 weeks' gestation, AKI has a significant short term and long-term impacts on renal health necessitating a long term follow up.

Progress in neonatal AKI has been slow, primarily due to under recognition of AKI by neonatologists and pediatricians and due to the lack of universally accepted diagnostic criteria. Several neonatal AKI definitions have been proposed in the past but more recently, the Kidney Diseases: Improving Global Outcomes (KDIGO) neonate modified classification is gaining more acceptance amongst neonatologists, pediatricians, and nephrologists. The neonate modified KDIGO is a tiered description of AKI based on changes in oliguria severity and serum creatinine (SCr) levels (5, 6). Moreover, the data on neonatal AKI from the Indian subcontinent is very small and regional risk profile predisposing newborn to develop AKI is not well-understood. Reported incidence of neonatal AKI in India ranges from 3.4 to 4.2% of all NICU admissions (7, 8). Understanding the practice patterns of neonatologists and pediatricians caring for neonates in India is important to optimize care and outcomes in neonatal AKI.

The objective of this study was to identify differences in physicians' (neonatologists and pediatricians) perception and practice variations of diagnosis, management, and follow-up of newborn infants with AKI in India after the publication of the AWAKEN study.

## Methods

### Study Design

The study followed a cross-sectional survey design. The target population for the survey was neonatologists and pediatricians caring for newborn infants. The study was conceptualized and developed by the Indian Neonatal Kidney Collaborative Study Group, which includes seven pediatric nephrologists and two neonatologists.

### Survey Instrument

The survey instrument was created on an online survey platform (Survey Monkey) by the Indian Neonatal Kidney Collaborative Study Group that included pediatric nephrologists and neonatologists. It was evaluated for both face and content validity by the investigators and approved by the Indian Society of Pediatric Nephrology. The survey was pilot tested on a group of neonatologists and pediatricians before distribution. Feedback from the pilot testing was used to modify and finalize questions. The survey instrument included questions referencing demographic data on providers, years of experience, level of neonatal care provided, type-severity-number of patients seen monthly (preterm infants, intubation with assisted ventilation, birth asphyxia/hypoxic ischemic encephalopathy), and availability of pediatric subspecialists including nephrologists. The survey instrument also included a common clinical scenario of AKI occurrence in preterm infants in the NICU (see below) and asked questions to elicit differences in physicians (neonatologists and pediatricians) perception and practice variations of diagnosis, management, and follow-up.

### Survey Distribution

The survey was administered electronically via email along with instructions and a hyperlink to the survey. The participants were notified of the voluntary nature of participation, confidentiality and non-compensation for participation. Lists of email addresses of neonatologists and neonatal practitioners were assembled by liaising with the Neonatology Chapter of Indian Academy of Pediatrics (IAP Neochap). The invitation was sent to 800 neonatologists and pediatricians nationwide in India during the month of January 2018. To encourage response rates, an additional survey reminder was emailed 1 month later.

### Statistical Analysis

Descriptive statistics was used to analyze data. A panel of one pediatric nephrologist (SKS) and two neonatologists (GA, SW) was formed to determine the optimal answers for the questionnaire of published literature or consensus.

## Results

### Participant Characteristics

Out of 800 correspondents, 257 completed the survey with response rate being 32.1%: out of which 52% were neonatologists and 48% were pediatricians. Amongst all the respondents, 29.5% worked in a teaching hospital and 39% in private practice, while 59% were attached to a level III NICU. The majority (71%) of respondents were between ages 30–50 years, 15% ≤ 30 and remaining 14% over 50 years. Twenty five percent of respondents had clinical experience of <3 years, 54% had 3–15 years' experience and 21% had >15 years' experience.

### Participant Exposure to Patient Variety and Resources

**Figure 1:**
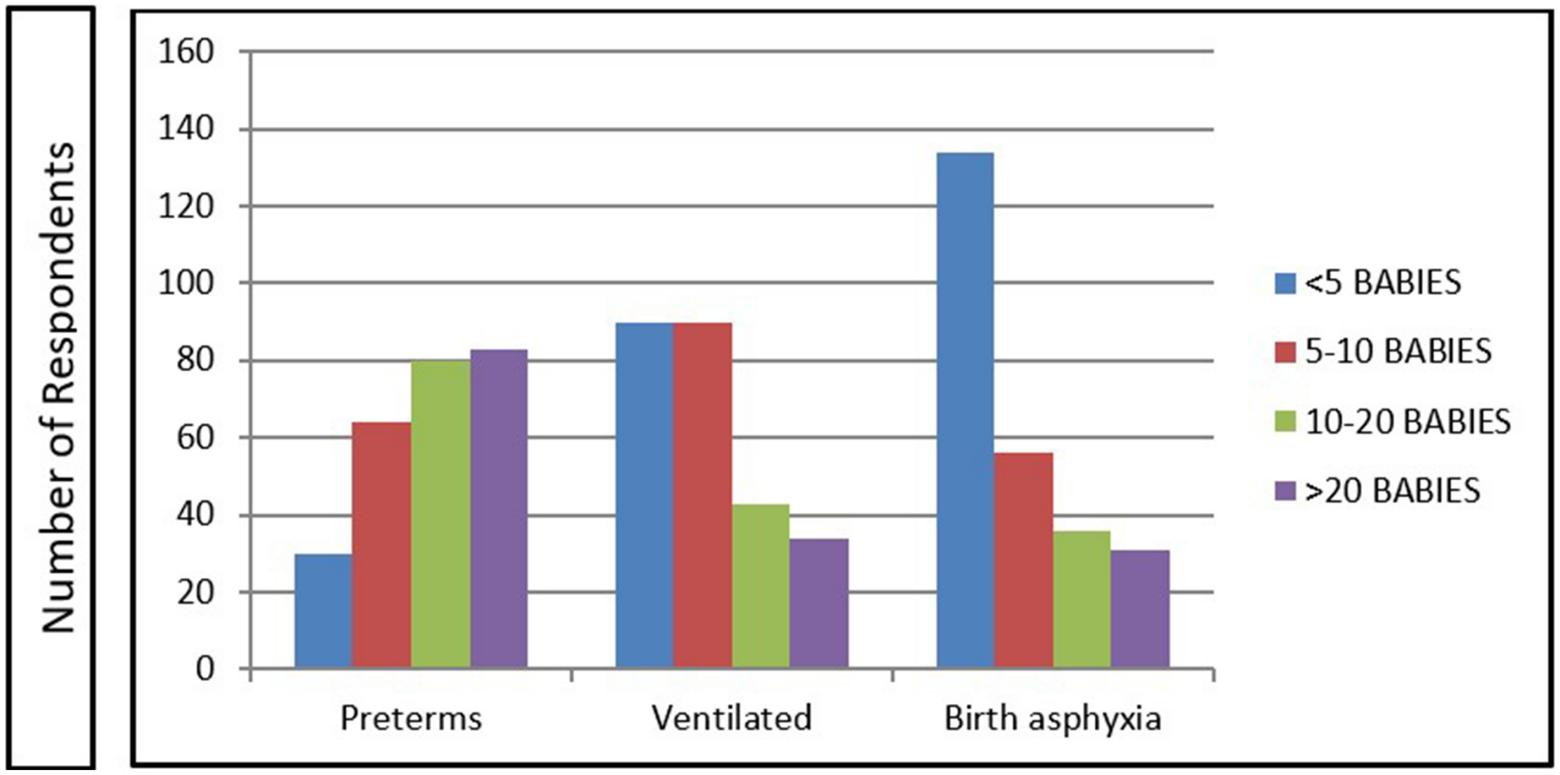
Newborn infants at risk for AKI seen by the respondents per month.

Figure 1 demonstrates the number of newborn infants at risk (preterm, ventilated, birth asphyxia) for AKI dealt by the respondents per month. Nearly two-third (60.3%) of the institutes of respondents had pediatric-neonatal surgery capabilities, whereas neonatal dialysis facilities were available for 11% and extracorporeal membrane oxygenation (ECMO) for only 3%. Only 31% of the respondents had the facility to access the services of a pediatric nephrologist.

### Perception and Practice Variations Regarding Diagnostic Criteria (Definitions) for Neonatal AKI

Table 1 demonstrates the perception of respondents in the incidence of neonatal AKI in the presence of various risk factors (prematurity, asphyxia, cardiac surgery, sepsis, medications). There was significant disagreement in the above perceptions and AKI decided by the expert panel. The panel notes, AKI in preterm infants was 41–60%, in asphyxia 21–40%, cardiac surgery 41–60%, sepsis 21–40%, and due to medications was 41–60%. Prematurity was considered to be a low risk factor for AKI by most of the respondents (81.7%) and only 2 (0.8%) respondents matched with the panel's opinion. More than 50% of respondents were unaware that cardiac surgery was a high-risk factor. The incidences of neonatal AKI due to the use of various medications was considered to be 41-60% by the expert panel but only 2 (0.8%) respondents had a similar response.

**Table 1:** Perception of respondents regarding the incidence of neonatal AKI in various clinical conditions.

	**Incidence of neonates developing AKI**				
**Clinical conditions**	**<20**	**21–40**	**41–60**	**>60**	**Not sure**
Preterm, *n* (%)	210 (81.7)	20 (7.8)	2 (0.8)	1 (0.4)	24 (9.3)
Asphyxia, *n* (%)	127 (49.4)	81 (31.5)	32 (12.5)	7 (2.7)	10 (3.9)
Cardiac surgery, *n* (%)	63 (24.5)	34 (13.2)	17 (6.7)	3 (1.1)	140 (54.5)
Sepsis, *n* (%)	133 (51.6)	71 (27.6)	34 (13.4)	7 (2.8)	12 (4.6)
Medications, *n* (%)	169 (65.8)	24 (9.4)	2 (0.8)	3 (1.1)	59 (22.9)

Familiarity with various diagnostic criteria of AKI was variable with most respondents being familiar (to least familiar) with pRIFLE (57.5%), AKIN (49.8%), and KDIGO (19.8%), whereas 16.3% were unaware of any criteria. However, in their own clinical practice to diagnose AKI, respondents most frequently (in decreasing order) used rise in serum creatinine (57%), urine output (51%), pRIFLE (39%), AKIN (28%), and KDIGO (11%). As per the expert panel, a pRIFLE diagnostic criterion was not useful in neonates whereas other criteria (AKIN, KDIGO, Urine output, rise in serum creatinine) could be used for diagnosing neonatal AKI.

### Practice Variations in Diagnosis, Management, and Follow-Up of 27-Week Preterm Infants With AKI

#### Clinical Scenario

A 27 weeks' gestation extreme preterm infant on empiric IV ampicillin, IV amikacin and on continuous positive airway pressure (CPAP) since birth was noted to have a significant systolic murmur associated with tachypnea and feeding intolerance on day 7. Following an echocardiogram confirmation of patent ductus arteriosus (PDA), 3 doses of IV indomethacin were administered day 8 onwards. Baseline serum creatinine (SCr) before indomethacin administration rose from 0.6 mg/dl (Day 7) to 1.0 mg/dl (Day 10). Subsequent SCr values ranged from 0.8 to 0.7 mg/dl (Days 11–15). Enalapril and furosemide were added on Day 15 for ongoing clinical symptoms. SCr rose to 2.1 mg/dl on Day 17. Enalapril and furosemide were stopped.

#### Assessment of Risk Factors for AKI

Respondents believed that AKI developed most likely (to least likely) after the administration of indomethacin (49%), prior to the administration of indomethacin (40%), and 11% believed that AKI occurred after the administration of furosemide and enalapril. Respondents graded the relative importance of risk factors in the development of AKI (in decreasing order of importance) as the administration of amikacin (26%), prematurity (21%), PDA (19%), administration of enalapril (17%), or furosemide (14%), and antenatal antibiotics (3%). The expert panel opined that the preterm infant developed AKI after adding indomethacin and all the factors described in the case scenario (Use of amikacin, presence of PDA, use of enalapril, use of furosemide, prematurity, use of antenatal antibiotics) could be considered as risk factors for developing AKI.

#### Evaluation Strategies

Respondents would involve a pediatric nephrologist with variable frequency on day 7 (34%), day 10 (44%), day 17 (10%), and the remaining 12%, only if the dialysis was deemed necessary. The majority of the responses agreed with the opinion of the panel who suggested involving pediatric nephrologist on day 10, when creatinine increased from 0.6 to 1.0 mg/dL. In reference to sending the first SCr in the scenario, 14% of respondents would send it within 12–24 h of age, 36% at 24–48 h, 36% at 48–72 h and remaining 14% would order SCr prior to initiating the Indomethacin. There, the respondents differed from the panel's view that the first serum creatinine should have been ordered before administering indomethacin.

#### Management Strategies

**Figure 2:**
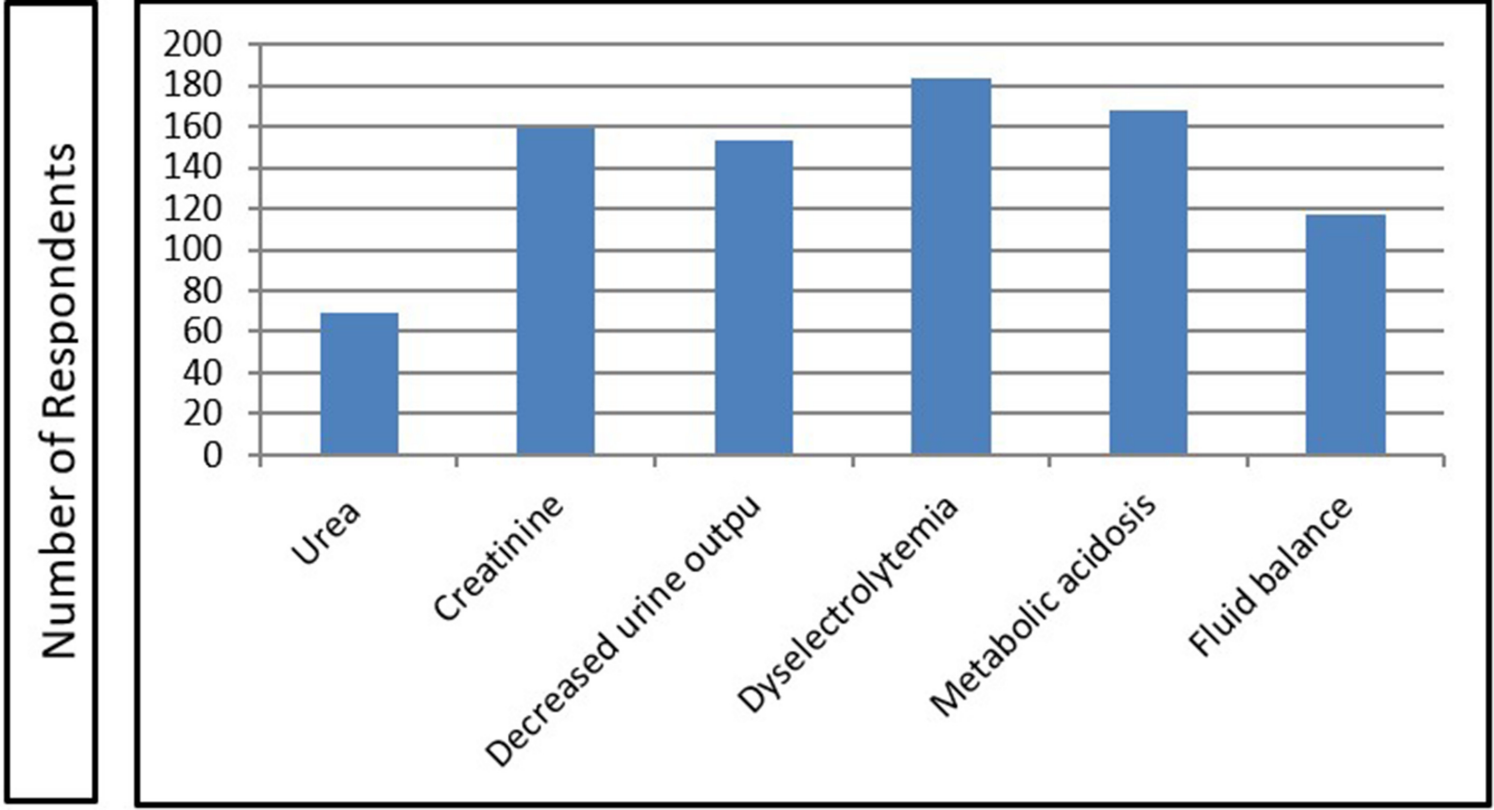
Criteria to initiate dialysis in the case scenario if renal function deteriorated.

Figure 2 demonstrates the criteria, which the respondents would use to initiate dialysis if the renal function in the case scenario deteriorated. Most common (to least common) criteria that the respondents would use to initiate dialysis were dyselectrolytemia (80%), metabolic acidosis (73%) and rising SCr (69%). The panel members had a consensus that urine output, dyselectrolytemia, metabolic acidosis, and fluid imbalance could be used as the criteria but urea and creatinine values should not be the criteria for initiation of dialysis. In such a scenario, majority of respondents would use peritoneal dialysis (84%), followed by hemodialysis (7%), and the rest (9%) would prefer continuous renal replacement therapy (CRRT), which almost matched with the view of panel members.

#### Follow-Up

Only 43% of respondents corroborated with the panel's view: that in the case scenario, the preterm infant was at risk for developing chronic kidney disease (CKD). In addition, over half (52%) of the respondents would not arrange a follow-up of the infant with a pediatric nephrologist, even though the panel suggested that all such infants should be followed up with a pediatric nephrologist.

## Discussion

In this first of its kind national survey in India of neonatologists and pediatricians caring for newborn infants in regard to neonatal AKI. The data demonstrated significant differences among practices for recognizing and defining AKI, renal function surveillance, nephrology consultation, dialysis options and follow up practices. The respondents demonstrated a variable degree of knowledge gaps of current definitions, early diagnosis, optimal management strategies, and follow up of newborn infants with AKI. This data may help in formulating future strategies to close these gaps.

The most common medical conditions in neonates associated with AKI are prematurity (42.2%) and congenital heart disease (CHD, 11.7%), with major independent risk factors being mechanical ventilation, hypervolemia, CHD and metabolic acidosis (9). In this survey, most respondents underestimated the risk of AKI due to various risk factors such as prematurity, asphyxia, sepsis, cardiac surgery, etc. There was a disagreement between the respondents, the views of the expert panel, and the published literature. In the study by Mian et al., the incidence of AKI in premature infants was 26% with incidence showing an inverse relation with the gestational age (GA) of the neonate. In infants between 22 and 25-weeks, AKI was seen in 65% of patients, while in infants between 26 and 28 weeks, this incidence was seen at 25% and a mere a 9% in infants between 29 and 32 weeks (10). In birth asphyxia, the incidence reported by Kaur et al. was 47.1% with incidence varying from 9.1% in moderate asphyxia to 56% in severe asphyxia (11). As per the study by Blinder et al., AKI incidence in neonates after cardiac surgery was 52% (12).

In order for these studies to be compared, standardized definition are required for neonatal AKI. This standard definition allows for the outcomes associated with AKI along with their epidemiology to be studied. To increase the accuracy of the results, AKI definitions have been changed [from pRIFLE to AKIN to KDIGO; (5, 6)]. More than half the respondents in our study were aware of pRIFLE criteria, which the expert panel considered least useful in neonates. Less than half the respondents were aware of AKIN or KDIGO criteria, which are the current standard criteria for defining neonatal AKI. Few respondents were aware about the new neonatal modified KDIGO classification. More than half used serum creatinine or urine output as the lone criteria. In a similar survey by Kent et al., nearly half of the neonatologists were unaware of the staged definitions of neonatal AKI (13). This study shows that there remains a gap in understanding regarding neonatal AKI and efforts at education are justified in the country.

A modifiable risk factor for AKI is the exposure to nephrotoxic medications. Rhone et al. have shown that during the stay of premature infants in the NICU, their exposure to nephrotoxic medications is 14 days on average (14). In our survey, most respondents felt that the risk of neonatal AKI due to various medications was <20%. This was in disagreement with the expert panel as they considered the incidence to be 41–60%. In the case scenario, only 49% respondents attributed AKI due to indomethacin use whereas only 26% felt that amikacin use can lead to AKI. This event brings up concerns as aminoglycosides and indomethacin have been shown to be frequently associated with AKI (15). Education in association with use of nephrotoxic medications usage in neonates can potentially minimize both the risk and incidence of AKI in neonates.

In recent years, renal replacement therapy has moved from being the last treatment option to a therapy designed to inhibit the effects of AKI in patients early (16). An interesting finding was that the good understanding between the respondents and the expert panel in response to the indications of dialysis and the mode of renal replacement therapy. Peritoneal dialysis was the most common mode while 9% considered CRRT as the first line of renal replacement therapy. The feasibility and technology available for doing CRRT in an extreme preterm infant needs to be considered.

There is strong evidence to convey that there is an increased risk of CKD with each episode of AKI. There is also evidence that extreme prematurity and exceedingly low birth weight are associated with CKD. However, there is currently no follow-up study reported that is large enough to evaluate the relationship of the risk of CKD and AKI in neonates. Small single-center studies have shown an increased risk of CKD in certain neonatal populations (17–19). In this survey, only half of the respondents were aware of the risk of CKD in preterm neonates and nearly the other half were unaware of the need to follow up with a pediatric nephrologist. A follow up of AKI at 3 months post discharge is recommended by the KDIGO guidelines. In this survey, only 31% had exposure to a pediatric nephrologist. This suggests that neonatologists/pediatricians may fail to diagnose AKI and lead to delayed consultations and treatments. Multiple studies have illustrated that lower mortality rates in adults is associated with early nephrology consultation (20, 21).

This study has several limitations including a small sample size, which may not be representative of the total cohort. Additionally, there was a very limited response rate which may have been due to the resource limitations, the lack of neonatology fellowship programs, and the small handful of practicing neonatologists in this country. Another limitation is that the results are subject to both survey bias and non-response error. Conceivably, physicians with interest in or previous experience in managing neonatal AKI were more likely to respond to this survey. As is the case with any survey, this one is also limited by its population set and may not be representative of the whole population.

In conclusion, this study demonstrates that there exists an important gap in knowledge about the effects of AKI in neonates. Increased efforts at education are crucial in order to improve clinical outcomes in the country. The development of guidelines and increase in education may potentially minimize the incidence and risk associated with neonatal AKI. Evaluating the long-term consequences of neonatal AKI and improving the management and its outcomes will require collaborations in both clinical care and research.

## Data Availability Statement

The raw data supporting the conclusions of this article will be made available by the authors, without undue reservation, to any qualified researcher.

## Author Contributions

SS, GA, SR, RJ, SW, and RoC helped in study design, carried out the survey, analyzed the data, and drafted the manuscript. SS, GA, RaC, NN, DK, RS, AI, RR, and SW supervised the collection, analysis of data, did critical revision, and finalization of the manuscript.

### Conflict of Interest

The authors declare that the research was conducted in the absence of any commercial or financial relationships that could be construed as a potential conflict of interest.

## References

[1] Abdulkader RC Liborio AB Malheiros DM. Histological features of acute tubular necrosis in native kidneys and long-term renal function. Ren Fail. (2008) 30:667–73. 10.1080/08860220802212460 18704814

[2] Coca SG Singanamala S Parikh CR. Chronic kidney disease after acute kidney injury: a systematic review and meta-analysis. Kidney Int. (2012) 81:442–48. 10.1038/ki.2011.379 22113526 PMC3788581

[3] Gouyon JB Guignard JP. Management of acute renal failure in newborns. Pediatr Nephrol. (2000) 14:1037–44. 10.1007/s004670050068 10975322

[4] Drukker A Guignard JP. Renal aspects of the term and preterm infant: a selective update. Curr Opinion Pediatr. (2002) 14:175–82. 10.1097/00008480-200204000-00006 11981287

[5] Jetton JG Askenazi DJ. Update on acute kidney injury in the neonate. Curr Opinion Pediatr. (2012) 24:191–6. 10.1097/MOP.0b013e32834f62d5 22227783 PMC5545784

[6] Jetton JG Guillet R Askenazi DJ Dill L Jacobs J Kent AL Assessment of worldwide acute kidney injury epidemiology in neonates (AWAKEN): design of a retrospective cohort study. Front Pediatr. (2016) 4:68 10.3389/fped.2016.00068 27486571 PMC4950470

[7] Aggarwal A Kumar P Chowdhary G Majumdar S Narang A. Evaluation of renal functions in asphyxiated newborns. J Trop Pediatr. (2005) 51:295–99. 10.1093/tropej/fmi017 16000344

[8] Bansal SC Nimbalkar AS Kungwani AR Patel DV Sethi AR Nimbalkar SM. Clinical profile and outcome of newborns with acute kidney injury in a level 3 neonatal unit in Western India. J Clin Diagn Res. (2017) 11:SC01–4. 10.7860/JCDR/2017/23398.9327 28511469 PMC5427395

[9] Duzova A Bakkaloglu A Kalyoncu M Poyrazoglu H Delibas A Ozkaya O. Etiology and outcome of acute kidney injury in children. Pediatr Nephrol. (2010) 25:1453–61. 10.1007/s00467-010-1541-y 20512652

[10] Mian AN Guillet R Ruck L Wang H Schwartz GJ. Acute kidney injury in premature, very low-birth-weight infants. J Pediatr Intensive Care. (2016) 5:69–78. 10.1055/s-0035-1564797 31110888 PMC6512402

[11] Kaur S Jain S Saha A Chawla D Parmar VR Basu S. Evaluation of glomerular and tubular renal function in neonates with birth asphyxia. Ann Trop Paediatr. (2011) 31:129–34. 10.1179/146532811X12925735813922 21575317

[12] Blinder JJ Goldstein SL Lee VV Baycroft A Fraser CD Nelson D. Congenital heart surgery in infants: effects of acute kidney injury on outcomes. J Thorac Cardiovasc Surg. (2012) 143:368–74. 10.1016/j.jtcvs.2011.06.021 21798562

[13] Kent AL Charlton JR Guillet R Gist KM Hanna M Samra A El Neonatal acute kidney injury: a survey of neonatologists' and nephrologists' perception and practice management. Am J Perintol. (2018) 35:1–9. 10.1055/s-0037-1604260 28709164

[14] Rhone ET Carmody JB Swanson JR Charlton JR. Nephrotoxic medication exposure in very low birth weight infants. J Matern Fetal Neonatal Med. (2014) 27:1485–90. 10.3109/14767058.2013.860522 24168068

[15] Akima S Kent A Reynolds GJ Gallagher M Falk MC. Indomethacin and renal impairment in neonates. Pediatr Nephrol. (2004) 19:490–3. 10.1007/s00467-003-1402-z 15007713

[16] Sutherland SM Alexander SR. Continuous renal replacement therapy in children. Pediatr Nephrol. (2012) 27:2007–16. 10.1007/s00467-011-2080-x 22366896

[17] Menon S Kirkendall ES Nguyen H Goldstein SL. Acute kidney injury associated with high nephrotoxic medication exposure leads to chronic kidney disease after 6 months. J Pediatr. (2014) 165:522–7.e2. 10.1016/j.jpeds.2014.04.058 24928698

[18] Abitbol CL Bauer CR Montané B Chandar J Duara S Zilleruelo G. Long-term follow-up of extremely low birth weight infants with neonatal renal failure. Pediatr Nephrol. (2003) 18:887–93. 10.1007/s00467-003-1186-1 12836091

[19] Bruel A Rozé JC Quere MP Flamant C Boivin M Roussey-Kesler G. Renal outcome in children born preterm with neonatal acute renal failure: IRENEO-a prospective controlled study. Pediatr Nephrol. (2016) 31:2365–73. 10.1007/s00467-016-3444-z 27335060

[20] Costa e Silva VT Liaño F Muriel A Díez R de Castro I Yu L. Nephrology referral and outcomes in critically ill acute kidney injury patients. PLoS ONE. (2013) 8:e70482. 10.1371/journal.pone.0070482 23936440 PMC3732261

[21] Meier P Bonfils RM Vogt B Burnand B Burnier M Referral patterns and outcomes in non-critically ill patients with hospital-acquired acute kidney injury. Clin J Am Soc Nephrol. (2011) 6:2215–25. 10.2215/CJN.01880211 21817132 PMC3359001

